# India’s Integrated Child Development Services programme; equity and extent of coverage in 2006 and 2016

**DOI:** 10.2471/BLT.18.221135

**Published:** 2019-02-25

**Authors:** Suman Chakrabarti, Kalyani Raghunathan, Harold Alderman, Purnima Menon, Phuong Nguyen

**Affiliations:** a Poverty, Health and Nutrition Division, The International Food Policy Research Institute, 2001 K Street NW, Washington DC, 20005, United States of America.

## Abstract

**Objective:**

To investigate coverage and equity of India’s Integrated Child Development Services programme across the continuum of care from pregnancy to early childhood, before and after the programme was expanded to provide universal access.

**Methods:**

The programme offers nutrition and health services to pregnant and lactating mothers and young children. We used data from nationally representative surveys in 2005–2006 and 2015–2016, including 36 850 mother–child pairs in 2006 and 190 804 in 2016. We assessed changes in the equity of use of programme services by socioeconomic quintile, caste, education and rural or urban residence. We used regression models to investigate the determinants of programme use.

**Findings:**

The mean proportion of respondents using programme services increased between 2006 and 2016, from 9.6% to 37.9% for supplementary food, 3.2% to 21.0% for health and nutrition education, 4.5% to 28% for health check-ups and 10.4% to 24.2% for child-specific services (e.g. immunization, growth monitoring). Wealth, maternal education and caste showed the largest positive associations with use of services. However, expansion in service use varied at the sub-national level. Although overall use had improved and reached marginalized groups such as disadvantaged castes and tribes, the poorest quintiles of the population were still left behind, especially in the largest states that carry the highest burden of undernutrition.

**Conclusion:**

India’s policy reforms have increased coverage of the programme at the national level, including for marginalized groups. With further scaling-up, the programme needs to focus on reaching households from the lowest socioeconomic strata and women with low schooling levels.

## Résumé

**Objectif:**

Étudier la couverture et l'équité du programme indien de services intégrés pour le développement de l'enfant (*Integrated Child Development Services*) tout au long du continuum de soins, depuis la grossesse jusqu'à la petite enfance, avant et après l'extension du programme visant à garantir un accès universel.

**Méthodes:**

Ce programme propose des services de nutrition et de santé aux femmes enceintes, aux femmes qui allaitent et aux jeunes enfants. Nous avons utilisé des données provenant d'enquêtes nationalement représentatives, réalisées en 2005–2006 et en 2015–2016, qui ont impliqué 36 850 paires mère-enfant en 2006 et 190 804 paires mère-enfant en 2016. Nous avons évalué les changements en matière d'équité dans l'utilisation de ces services par quintile socioéconomique, caste, niveau d'éducation et résidence rurale ou urbaine. Nous avons utilisé des modèles de régression pour étudier les déterminants de l'utilisation du programme.

**Résultats:**

La proportion moyenne de personnes interrogées utilisant les services du programme a augmenté entre 2006 et 2016, en passant de 9,6% à 37,9% pour la fourniture de suppléments alimentaires, de 3,2% à 21,0% pour les services d'éducation sur la santé et la nutrition, de 4,5% à 28% pour les bilans de santé et de 10,4% à 24,2% pour les services spécifiques à l'enfant (ex.: vaccination, surveillance de la croissance). Le niveau de revenus, le niveau d'éducation de la mère et la caste correspondent aux associations les plus largement positives avec l'utilisation des services. Néanmoins, l'augmentation de l'utilisation des services a varié au niveau sous-national. Même si l'utilisation générale a augmenté et que les services ont atteint des groupes marginalisés, tels que les castes et les tribus les plus défavorisées, les quintiles les plus pauvres de la population sont restés laissés pour compte, notamment dans les plus grands états, qui sont les plus lourdement frappés par la dénutrition.

**Conclusion:**

En Inde, les réformes des politiques ont permis d'obtenir une meilleure couverture au niveau national, y compris pour les groupes marginalisés. Pour ses prochaines extensions, le programme devra cibler les ménages appartenant aux couches socioéconomiques les plus pauvres et les femmes ayant un niveau d'éducation bas.****

## Resumen

**Objetivo:**

Investigar la cobertura y la equidad del Programa de servicios integrados de desarrollo infantil de la India a través de la atención continua desde el embarazo hasta la infancia temprana, antes y después de que el programa se ampliara para proporcionar acceso universal.

**Métodos:**

El programa ofrece servicios de nutrición y salud a madres embarazadas y lactantes y a niños pequeños. Se utilizaron datos de encuestas representativas a nivel nacional de 2005-2006 y 2015-2016, incluidas 36 850 parejas de madres e hijos en 2006 y 190 804 en 2016. Se evaluaron los cambios en la equidad de uso de los servicios del programa por quintil socioeconómico, casta, educación y residencia rural o urbana. Se utilizaron modelos de regresión para investigar los determinantes del uso del programa.

**Resultados:**

La proporción media de encuestados que utilizan los servicios del programa aumentó entre 2006 y 2016, del 9,6 % al 37,9 % en el caso de los alimentos suplementarios, del 3,2 % al 21,0 % en el de la educación sanitaria y nutricional, del 4,5 % al 28 % en el de los reconocimientos médicos y del 10,4 % al 24,2 % en el de los servicios específicos para niños (por ejemplo, inmunización o vigilancia del crecimiento). La riqueza, la educación materna y la casta mostraron las mayores asociaciones positivas con el uso de los servicios. Sin embargo, la expansión del uso de servicios varió a nivel subnacional. Aunque el uso general ha mejorado y ha llegado a grupos marginados como las castas y tribus desfavorecidas, los quintiles más pobres de la población siguen quedando rezagados, especialmente en los estados más grandes que soportan la mayor carga de desnutrición.

**Conclusión:**

Las reformas políticas de la India han aumentado la cobertura del programa a nivel nacional, incluso para los grupos marginados. Con una mayor ampliación, el programa debe centrarse en llegar a los hogares de los estratos socioeconómicos más bajos y a las mujeres con bajos niveles de escolaridad.

## ملخص

**الغرض:**

التحقق من عدالة ومدى تغطية برنامج الهند لخدمات تنمية الطفل، وذلك من خلال استمرار الرعاية من الحمل وحتى الطفولة المبكرة، قبل وبعد توسيع البرنامج لتوفير إمكانية الوصول الشامل.

**الطريقة:**

يقدم البرنامج خدمات التغذية والصحة للأمهات الحوامل والمرضعات والأطفال الصغار. قم باستخدام البيانات من عمليات المسح التمثيلي الوطنية في الفترة 2005 إلى 2006، والفترة 2015 إلى 2016، بما في ذلك 36850 زوجا من الأمهات والأطفال في عام 2006، و190804 في عام 2016. وقمنا بتقييم التغيرات التي طرأت على العدالة في الحصول على خدمات البرنامج من بواسطة نسبة الخمس من الفئات الاجتماعية والاقتصادية، والطبقات السكانية، والتعليم، والإقامة الريفية أو الحضرية. لقد استخدمنا نماذج التحوف لاستقصاء محددات استخدام البرنامج.

**النتائج:**

ازدادت النسبة المتوسطة للمشاركين الذين يستخدمون خدمات البرنامج بين عامي 2006 و2016، من 9.6٪ إلى 37.9٪ للأغذية التكميلية، ومن 3.2٪ إلى 21٪ للتعليم الصحي والتغذية، ومن 4.5٪ إلى 28٪ للفحوصات الصحية، ومن 10.4٪ إلى 24.2% للخدمات الخاصة بالأطفال (مثل التحصين ومراقبة النمو). وأظهرت الثروة، وتعليم الأمهات، والطبقات الاجتماعية، أكبر ارتباطات إيجابية باستخدام الخدمات. ومع ذلك ، تنوع التوسع في استخدام الخدمة على المستوى دون الوطني. وعلى الرغم من أن الاستخدام الإجمالي قد تحسن ووصل إلى الفئات المهمشة مثل الطبقات والقبائل المحرومة، فإن أفقر النسبة الخمسية من السكان ما زالت منسية، وبخاصة في أكبر الولايات التي تتحمل العبء الأكبر لنقص التغذية.

**الاستنتاج:**

أدت إصلاحات السياسة في الهند إلى زيادة تغطية البرنامج على المستوى الوطني، بما في ذلك الفئات المنسية. ومع المزيد من التوسع، يحتاج البرنامج للتركيز على الوصول إلى الأسر من أدنى المستويات الاجتماعية والاقتصادية، والسيدات من المستويات التعليمية المنخفضة.

## 摘要

**目的:**

旨在研究印度儿童综合发展服务项目在扩大规模、全面普及前后，在怀孕初期至幼儿期的连续护理过程中该项目的覆盖范围和公平性。

**方法:**

该项目为孕妇、哺乳期母亲和幼儿提供营养和健康服务。我们使用了 2005–2006 年和 2015–2016 年全国性调查的数据，其中包括 2006 年的 36，850 对母子和 2016 年的 190，804 对母子。我们通过社会经济五分位数、种姓、教育和农村或城市居民对项目服务使用的公平性变化进行了评估。我们使用回归模型来研究项目使用的决定因素。

**结果:**

研究表明在 2006 年至 2016 年期间，使用项目服务的受访者的平均比例有所增加，辅食方面从 9.6％ 上升至 37.9％，健康和营养教育方面从 3.2％ 上升 至 21.0％，健康检查方面从 4.5％ 上升至 28％，而且儿童特定服务（例如免疫接种、生长发育监测）由 10.4％ 上升至 24.2%。财富、孕产妇受教育程度和种姓与服务使用呈现最大程度上的正相关。然而，服务使用的扩大在地方各级间差异显著。虽然总体使用情况得以改善，且已触及边缘化群体（例如弱势种姓和部落），但最贫困的五分之一人口仍然落后，尤其是在营养不良、负担最重的各邦。

**结论:**

印度的政策改革扩大了该项目在国家层面的覆盖范围，同时也囊括了边缘化群体。随着规模的进一步扩大，该项目需要聚焦于触及社会经济阶层最底层的家庭和受教育程度较低的妇女。****

## Резюме

**Цель:**

Исследование охвата и равноправного доступа к комплексным услугам по развитию ребенка в Индии в рамках всей цепочки услуг здравоохранения: от беременности до дошкольного возраста; до и после того, как программа стала общедоступной.

**Методы:**

Программа предлагает питание и медицинское обслуживание беременным женщинам и кормящим матерям, а также маленьким детям. Авторы использовали данные национальных репрезентативных опросов, проведенных в 2005–2006 и 2015–2016 годах, в которых участвовали 36 850 пар матерей с детьми в 2006 году и 190 804 пары в 2016 году. Были оценены изменения в равенстве использования услуг программы в зависимости от социально-экономического квинтиля, кастовой принадлежности, образования и района проживания в сельской или городской местности. Для исследования детерминант использования программы использовались регрессионные модели.

**Результаты:**

Средняя пропорция респондентов, пользующихся услугами программы для получения дополнительного питания, выросла с 2006 по 2016 год с 9,6 до 37,9%, с 3,2 до 21,0% возросло количество тех, кто использовал программу для получения знаний о здоровье и питании, с 4,5 до 28% возросло количество тех, кто проходил медицинский осмотр в рамках программы, с 10,4 до 24,2% — количество потребителей услуг, ориентированных на детей (прививки, контроль развития). Наибольший рост использования услуг наблюдался по показателям уровня благосостояния, образования матери и кастовой принадлежности. Однако на субнациональном уровне распространенность использования услуг варьировалась. Несмотря на то что показатели общего использования программы улучшились и она стала доступнее маргинализированным группам, таким как низшие касты и племена, беднейшие квинтили населения все еще не имеют доступа к ней, особенно в крупнейших штатах, на которые приходятся самые высокие показатели недоедания.

**Вывод:**

Политика реформ, проводимая в Индии, расширила охват программой на национальном уровне, включая маргинализированные группы населения. При дальнейшем расширении масштабов программа должна стать доступной для беднейших социально-экономических слоев населения и для женщин с низким уровнем образования.

## Introduction

In 2013, reviews of effective nutrition interventions estimated that scaling-up a set of proven nutrition-specific interventions could reduce stunting globally by 20% and reduce child mortality by 15%.^1^ Other modelling exercises have attempted to estimate the potential impact of scaling-up key interventions on progress towards sustainable development goals or on economic growth.^2^^,^^3^ Few studies, however, have examined how programmes expand to achieve interventions at scale. Fewer still have assessed the extent to which programme expansion reaches the most vulnerable populations. However, it is the juxtaposition of coverage and efficacy that explains progress in reducing malnutrition or its absence.

Nutrition programmes that include targeted food assistance have been implemented at large-scale worldwide for several years, such as the Women, Infants and Children Programme in the United States of America^4^ and the *Oportunidades* programme in Mexico.^5^ Studies of targeted programmes have documented respondents’ characteristics and enrolment in and coverage of the programme,^5^ but less is known about factors affecting uptake of universally offered programme services. India provides a case study to examine the scale-up and uptake of a large-scale, universal, food-assisted maternal and child nutrition programme.^6^

India launched its Integrated Child Development Services programme in 1975^7^ and expanded it to all states in 2000. However, services were patchy throughout the early 2000s.^8^ In 2006, India’s Supreme Court ruled that the programme was to be offered universally and, soon after, the government expanded the availability of programme services across India, with a goal of ensuring about 1.4 million programme centres across the country. The programme now serves about 82 million children younger than 6 years and over 19 million pregnant women and lactating mothers.^6^^,^^9^ Services currently include take-home supplementary food and hot cooked meals, health and nutrition education, and health check-ups delivered at rural child-care centres (called *anganwadi*) or at home.^7^ Immunizations, growth monitoring and pre-school care services are also available for children at the centres.^10^

There is mixed evidence on the coverage and effectiveness of India’s programme on nutrition and related outcomes. Studies from the 1990s generally found programme placement that was skewed towards the well-off districts and no effects on anthropometric outcomes.^11^^,^^12^ Studies from the following decade reported positive impacts, but with important caveats. For instance, a study based on 2005–2006 data found that girls who received supplementary food from the services were on average taller.^13^ However, only 6% (329) of 5364 girls aged 0 to 2 years and 14% (1113) of 7951 girls aged 3 to 5 years in that study received the supplementary food, despite the fact that over 90% of villages (3522/3849) had an integrated child development centre. This result raised questions about the factors influencing programme uptake.^13^ These studies preceded major reforms in the services between 2006 and 2009.^7^ The reforms included greater financial outlay from the central government and provision of supplementary food in a rights-based framework.^14^

Most research on the delivery of India’s Integrated Child Development Services programme in the period after the reforms has focused on performance in implementation of the programme by states,^10^ with limited evidence on individual and household uptake or use of the programme. In view of these gaps, we investigated changes in the use of the services over the continuum of care from pregnancy up to early childhood between 2006 and 2016. We assessed equity gaps and factors associated with use of services. Our analysis has policy implications for India, but also offers global lessons to other countries embarking on scaling-up integrated programmes to address maternal and child health, nutrition and child development.

## Methods

### Data sources

We used data from two rounds of India’s national family health surveys, in 2005–2006^15^ and 2015–2016.^16^ These cross-sectional surveys follow a systematic, multi-stage stratified sampling design, covering all states and union territories in India. While the 2006 round was representative at the state level, the 2016 survey was representative at both the state and district levels. We use data from the data sets for households (109 041 in 2006 and 601 509 in 2016), women (138 592 in 2006 and 749 344 in 2016) and children (36 850 in 2006 and 190 804 in 2016).

### Outcome variables

Our primary outcomes were receipt of programme interventions among pregnant and lactating women aged 15 to 49 years and their children aged 0 to 59 months. We restricted analyses to last-born children to minimize recall bias. We analysed 12 outcomes of the programme services for mothers and children. We grouped them into four types of services (supplementary food, counselling on nutrition, health check-ups and early childhood services), offered over three phases in the care continuum (pregnancy, lactation and early childhood). Detailed definitions of the indicators are provided in the data repository.^17^

For the analyses of inequity and determinants of use of services, we constructed composite indicators to represent receiving services during all three phases of the care continuum. Mathematically, for each service k, the composite indicator *Y_k_* was defined as:
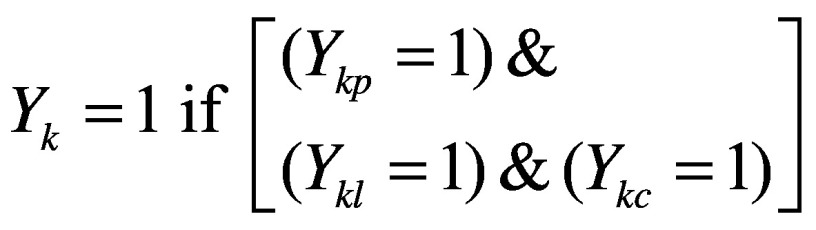
(1)Where *Y_kx_*denotes receiving service *k* in period *x*, and *x* can be pregnancy (*p*), lactation (*l*) or early childhood (*c*). We analysed receipt of the following services by mother–child pairs: supplementary food, counselling on nutrition practices and health check-ups. For services that are only provided during early childhood (immunizations, pre-school education and growth monitoring services) the composite indicator is simply an indicator for the child having received the service.

We also examine the changes in the frequency of receiving supplementary food in the 12 months preceding the survey. This question is only available for the child and does not clarify whether the supplementary food received is take-home rations or daily food at the rural child-care centres, so the full amount of food received is uncertain. Therefore, we report on this question, but did not include it in the composite indicator.

### Explanatory variables

Household level variables included household size, socioeconomic status, religion, caste category, urban or rural residence and access to health insurance. The socioeconomic status score was constructed using a factor analysis of multiple variables including water source, toilet type, materials used in dwellings, and ownership of a house, land, livestock and durable assets.^18^ Mother- and child-specific variables included the mother’s age and education, and the child’s sex, age and birth order.

### Statistical analysis

We tested changes in outcomes and determinants from 2006 to 2016 using regression models. Equity analyses were conducted for the 12 individual outcome variables by socioeconomic status quintiles at the national level, for residential areas, castes and maternal education for the two survey rounds, adjusting for sample probability weights. We plotted changes in the services received within socioeconomic status quintiles for these categories.

We used multivariate logistic regression models to determine the association between explanatory variables and each of the four composite indicators. To examine changes over time, we conducted the regressions for 2006 and 2016 separately, with clustering of standard errors within states. Dummy variables for the 35 Indian states were included in all specifications.

Thus, for household *h* in states in time *t*, we estimated:
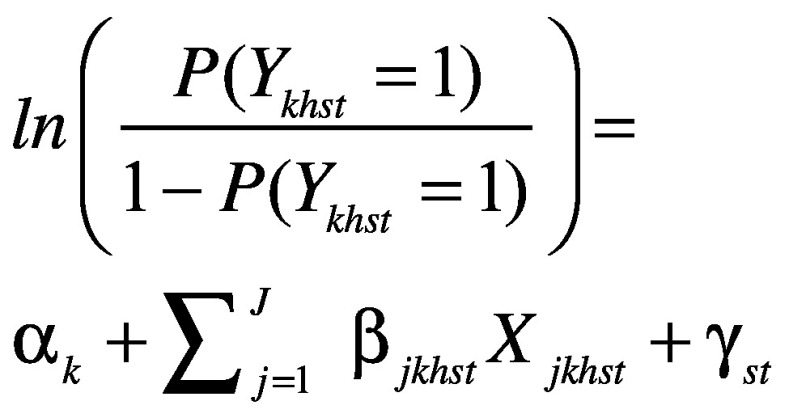
(2)Where *P *(*Y_khst_* = 1) denotes the probability that the composite indicator *Y_k_* took on value 1 for household *h* in state *s* in time *t*. We controlled for *J *covariates at the household level, as listed above under explanatory variables, and for state-fixed effects, γ*_st_*. Regressions were run separately for each composite indicator *k*.

For robustness checks, we conducted the regression analyses individually for all 12 services. Since different services are provided by child’s age group, we also examined the summary statistics for service coverage by age (< 6 months, 6 to 35 months and 36 to 59 months). We adjusted our models for state-specific performance using an index of programme performance developed using a survey from 2013–2014.^10^ The performance data covered programme infrastructure, knowledge and service provision of workers at rural child-care centres, and annual expenditure per child.^19^^,^^20^ Finally, we conducted separate regression analyses for two states with the highest burden of undernutrition in India (Uttar Pradesh and Bihar) to investigate if determinants differed compared with national-level estimates.^21^

### Results

Several individual and household characteristics changed appreciably between the two rounds of national family health surveys (Table 1**)**. The socioeconomic status distribution shifted towards wealthier quintiles, as poorer households acquired assets in the intervening years. Health insurance coverage increased from a mean of 3.7% (95% confidence interval, CI: 3.4 to 4.1) in 2006 to 24.3% (95% CI: 23.9 to 24.7) in 2016. Mothers’ education also improved, with the mean proportion of respondents reporting no schooling falling by nearly 20 percentage points, from 46.3% (95% CI: 45.1 to 47.6) to 27.0% (95% CI: 26.6 to 27.4). The distribution of other characteristics in the samples, including religious and caste composition, family size and urban residence, were similar across rounds.

**Table 1:** Characteristics of samples in the study of coverage and equity of the Integrated Child Development Services programme in India, 2006 and 2016

Characteristics	Year, mean value (95% CI)	
	2006 (*n* = 36 850)	2016 (*n* = 190 804)
**Household**		
Family size, no.	6.7 (6.6 to 6.7)	6.3 (6.3 to 6.3)
Socioeconomic status index,^a^ %		
Quintile 1 (poorest)	39.1 (37.9 to 40.3)	16.5 (16.2 to 16.9)
Quintile 2	22.4 (21.7 to 23.2)	18.4 (18.1 to 18.7)
Quintile 3	15.4 (14.8 to 16.01)	19.9 (19.6 to 20.3)
Quintile 4	12.8 (12.2 to 13.5)	22.0 (21.6 to 22.3)
Quintile 5 (richest)	10.3 (9.6 to 10.9)	23.2 (22.8 to 23.7)
Religion, %		
Hindu	78.9 (77.4 to 80.4)	78.8 (78.2 to 79.4)
Muslim	16.3 (14.8 to 17.8)	16.1 (15.5 to 16.6)
Christian	2.1 (1.8 to 2.4)	2.2 (2.0 to 2.3)
Caste categories, %		
Scheduled castes	19.9 (18.9 to 21.0)	21.3 (20.8 to 21.8)
Scheduled tribe	9.3 (8.4 to 10.2)	10.2 (9.9 to 10.5)
Other backward classes	40.3 (38.9 to 41.7)	43.0 (42.4 to 43.5)
Urban residence, %	27.1 (25.1 to 29.0)	30.0 (29.2 to 30.9)
Having health insurance, %	3.7 (3.4 to 4.1)	24.3 (23.9 to 24.7)
**Mother**		
Age, years	26.6 (26.5 to 26.7)	27.0 (27.0 to 27.1)
Education,^b^ %		
No schooling	46.3 (45.1 to 47.6)	27.0 (26.6 to 27.4)
Primary school	13.9 (13.4 to 14.5)	13.1 (12.9 to 13.4)
Secondary school	27.7 (26.8 to 28.6)	36.4 (36.0 to 36.9)
High school or higher	12.1 (11.4 to 12.8)	23.5 (23.0 to 23.9)
**Child**		
Sex (female), %	45.4 (44.7 to 46.1)	44.8 (44.5 to 45.2)
Age, months	24.0 (23.8 to 24.3)	25.1 (25.0 to 25.2)
Birth order, %		
First	24.6 (30.0 to 25.3)	31.8 (31.5 to 32.2)
Second	29.5 (28.7 to 30.2)	35.6 (35.2 to 36.0)
Third or more	45.9 (45.0 to 46.8)	32.6 (32.2 to 33.0)

CI: confidence interval.

^a^ Index of socioeconomic status was constructed by factor analysis using: household access to improved drinking water, improved latrine, clean cooking fuel, electricity, ownership of a house and land, housing materials for floor, roof and wall, and possession of 15 assets (including a mattress, pressure cooker, chair, bed, table, fan, television, sewing machine, phone, computer, refrigerator, watch, bicycle, motorbike and car) and livestock (cow, goat and chicken).

^b^ Education categories were grouped as follows for total number of years of education attainted by the individual: no schooling (0 years), primary school (1 to 5 years), secondary school (6 to 10 years) and high school or higher (≥ 11 years).

Notes: *n* is the total number of respondents. We analysed data from India’s national family health surveys in 2005–2006^15^ and 2015–2016.^16^ These cross-sectional surveys follow a systematic, multistage stratified sampling design, covering all states and union territories in India. Number of respondents for the mother’s age were 33 595 (2005–2006) and 174 050 (2015–2016) and for the child’s age were 35 321 (2005–2006) and 183 292 (2015–2016).

The receipt of services by mother–child pairs from pregnancy through to early childhood increased significantly between 2006 and 2016 (Table 2). The increase was especially noticeable for supplementary food, increasing nearly threefold from 9.6% of respondents (95% CI: 9.0 to 10.3) in 2006 to 37.9% (95% CI: 37.4 to 38.4) in 2016. Use of health check-ups increased 23.5 percentage points and health and nutrition education increased 17.9 percentage points. These patterns were consistent for the child age subgroups (data repository).^17^ The frequency of receiving supplementary food also improved, with a nearly 8 percentage point increase for children who received food monthly (Table 2) from 19.1% of children (95% CI: 17.83 to 20.39) to 27.6% (95% CI: 27.07 to 28.04).

**Table 2:** Trends in use of the Integrated Child Development Services programme among pregnant and lactating women and their children aged 0 to 59 months in India, 2006 and 2016

Indicator	2006			2016		Change, % point
	No. of respondents	% (95% CI)^a^		No. of respondents	% (95% CI)^a^	
**During pregnancy^b^**						
Supplementary food	6474	18.7 (17.7 to 19.6)		100 391	52.6 (52.1 to 53.2)	34.0
Health and nutrition education	3287	10.0 (9.3 to 10.7)		70 493	39.3 (38.8 to 39.8)	29.3
Health check-ups	3501	11.2 (10.5 to 12.0)		79 550	43.8 (43.3 to 44.3)	32.6
**During lactation^b^**						
Supplementary food	5334	14.7 (13.8 to 15.5)		90 752	47.8 (47.3 to 48.3)	33.2
Health and nutrition education	2464	7.2 (6.6 to 7.8)		62 493	35.9 (34.6 to 35.6)	27.9
Health check-ups	2392	7.5 (6.9 to 8.1)		66 761	37.2 (36.6 to 37.7)	29.7
**During childhood^b^**						
Take-home rations or hot cooked meals	7786	22.1 (21.1 to 23.1)		95 751	51.7 (51.2 to 52.3)	29.6
Immunizations	5478	18.3 (17.3 to 19.3)		76 019	43.2 (42.7 to 43.7)	24.9
Health check-ups	4255	14.2 (13.3 to 15.0)		74 904	42.7 (42.1 to 43.2)	28.5
Early childhood care or preschool education	9424	28.1 (27.0 to 29.3)		64 147	35.8 (35.4 to 36.3)	7.7
Child was weighed	4879	15.7 (14.8 to 16.6)		79 291	44.6 (44.1 to 45.1)	28.9
Mother received counselling after child was weighed	2423	7.9 (7.2 to 8.5)		49 430	28.6 (28.1 to 29.0)	20.7
**Integrated child development services in all periods^b^**						
Supplementary food	3364	9.6 (9.0 to 10.3)		68 883	37.9 (37.4 to 38.4)	28.3
Health and nutrition education	967	3.2 (2.8 to 3.5)		35 630	21.0 (20.6 to 21.5)	17.9
Health check-ups	1293	4.5 (4.0 to 4.9)		48 197	28.0 (27.5 to 28.5)	23.5
Child-specific services^c^	3068	10.4 (9.6 to 11.1)		41 929	24.2 (23.8 to 24.7)	13.9
**Frequency of receipt of supplementary food in previous 12 months^d^**						
Never	1745	22.2 (20.4 to 23.9)		10 527	10.3 (10.0 to 10.6)	−11.8
Daily	2649	30.8 (29.0 to 32.5)		31 573	35.5 (34.9 to 36.1)	4.7
Weekly	1723	17.8 (16.5 to 19.0)		25 407	21.8 (21.3 to 22.3)	4.0
Monthly	2167	19.1 (17.8 to 20.4)		31 372	27.6 (27.1 to 28.0)	8.4
Less than monthly	1247	9.5 (8.6 to 10.4)		7 399	4.6 (4.4 to 4.8)	−4.9

CI: confidence interval.

^a^ Percentages and confidence intervals were adjusted using sampling weights.

^b^ The total number of respondents were 36 850 and 190 804 in 2006 and 2016, respectively.

^c^ Immunizations, pre-school education and growth monitoring.

^d^ The total number of respondents were 9592 and 106 574 in 2006 and 2016, respectively.

**Fig. 1:**
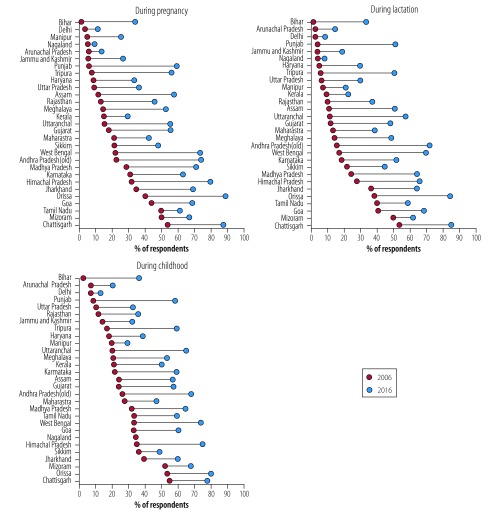
Trends in coverage of supplementary food in the Integrated Child Development Services programme during pregnancy, lactation and childhood across states of India, 2006 and 2016

Note: The total number of respondents were 36 850 and 190 804 in 2006 and 2016, respectively.

At the sub-national level there was substantial variation in the expansion of service use across the two survey rounds (Fig. 1). With some exceptions (Tamil Nadu, Odisha, Chhattisgarh and Jharkhand), the coverage of supplementary food during pregnancy and lactation was < 25% in most states in 2006. By 2016, this had improved in almost all states. The state-wise coverage of supplementary food during childhood was higher than in the other two categories, and coverage increased in all states by 2016, with increases to ≥ 50% in many of the central and southern states.

**Fig. 2:**
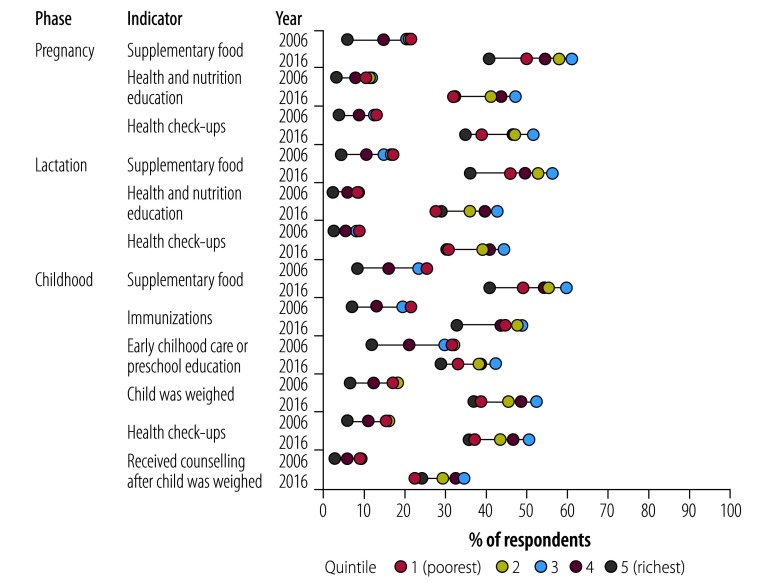
Socioeconomic status and use of the Integrated Child Development Services programme among pregnant and lactating women and their children in India, 2006 and 2016

Notes: The total number of respondents were 36 850 and 190 804 in 2006 and 2016, respectively. Index of socioeconomic status was constructed by factor analysis using: household access to improved drinking water, improved latrine, clean cooking fuel, electricity, ownership of a house and land, housing materials for floor, roof and wall, and possession of 15 assets (including a mattress, pressure cooker, chair, bed, table, fan, television, sewing machine, phone, computer, refrigerator, watch, bicycle, motorbike and car) and livestock (cow, goat and chicken).

Fig. 2 plots the mean percentage of respondents using various services in both 2006 and 2016, disaggregated by wealth quintile. Within a given year, a higher spread of dots indicates greater inequality in use. Use of the services did not grow uniformly. Although use expanded for the poorest quintile across all services, expansion was lower for quintiles 2, 3 and 4. In 2006, the lowest quintile had higher use than other quintiles, but by 2016, use by quintiles 2, 3 and 4 had expanded.

Use of the four categories of services among pregnant and lactating women and their children by respondents’ characteristics are shown in Table 3. Factors associated with use of services also changed over time (Table 4 and Table 5). In 2006, quintile 5 was the only quintile where receipt of counselling on nutrition was significantly lower than quintile 1 (odds ratio, OR: 0.43, 95% CI: 0.22 to 0.81). However, by 2016 use of nutrition counselling by quintile 5 was similar to quintile 1 (OR: 1.10, 95% CI: 0.97 to 1.25). Quintiles 2, 3 and 4 were significantly more likely to receive counselling services (OR: 1.13, 95% CI: 1.06 to 1.20; OR: 1.29, 95% CI: 1.16 to 1.44; and OR: 1.27, 95% CI: 1.12 to 1.43, respectively) than either quintiles 1 or 5. Similar findings were observed for health check-ups. For supplementary food only quintile 3 was significantly more likely than the poorest quintile to receive services in 2016 (OR: 1.17, 95% CI: 1.05 to 1.29). All coefficients for wealth quintiles in 2016 were significantly different from the corresponding ones in 2006.

**Table 3:** Use of the Integrated Child Development Services programme among pregnant and lactating women and their children in India, 2006 and 2016, by respondents’ characteristics

Characteristics	No. (%) of respondents using service^a^										
	Food supplements^b^			Counselling on nutrition^b^			Health check-ups^b^			Child-specific services^c^	
	2006	2016		2006	2016		2006	2016		2006	2016
**Household**											
Socioeconomic status index^d^											
Quintile 1 (poorest)	1277 (11.7)	10 699 (34.8)		345 (3.4)	4 539 (14.8)		528 (5.1)	6 685 (21.5)		1 161 (11.3)	6 232 (20.4)
Quintile 2	800 (10.9)	13 786 (41.7)		236 (3.6)	6 701 (21.6)		325 (5.2)	9 361 (29.4)		775 (12.1)	8 122 (24.9)
Quintile 3	594 (9.7)	15 741 (45.5)		195 (3.7)	8 421 (26.2)		236 (4.9)	11 236 (34.5)		577 (11.9)	9 590 (28.8)
Quintile 4	436 (6.4)	15 421 (39.7)		126 (2.4)	8 577 (24.3)		134 (3.0)	11 229 (31.3)		358 (7.6)	9 676 (27.1)
Quintile 5 (richest)	241 (2.2)	13 236 (28.4)		61 (1.0)	7 392 (17.5)		63 (1.1)	9 686 (22.7)		187 (3.8)	8 309 (19.8)
Religion											
Hindu	2606 (10.4)	53 740 (39.4)		790 (3.4)	28 752 (22.1)		1 095 (5.0)	38 951 (29.4)		2 562 (11.5)	33 784 (25.5)
Muslim	223 (4.9)	7 483 (29.9)		75 (1.6)	3 488 (15.5)		73 (1.6)	4 880 (21.3)		230 (4.9)	4 510 (17.8)
Christian	398 (12.0)	4 784 (38.6)		72 (4.0)	2 027 (21.9)		73 (4.6)	2 543 (28.4)		163 (8.2)	1 987 (21.8)
Other	137 (12.8)	2 876 (40.9)		30 (3.3)	1 363 (22.3)		52 (6.1)	1 823 (28.2)		113 (12.7)	1 648 (27.6)
Caste categories											
Scheduled castes	696 (12.2)	14 412 (42.8)		219 (4.1)	7 543 (23.8)		288 (5.4)	10 193 (31.4)		682 (12.2)	8 967 (27.5)
Scheduled tribe	975 (22.4)	15 799 (50.4)		235 (7.2)	7856 (27.6)		347 (11.3)	10 682 (38.3)		696 (22.4)	8 856 (32.2)
Other backward classes	1054 (8.7)	26 011 (35.9)		345 (3.0)	13 766 (19.7)		455 (4.2)	18 759 (26.2)		1077 (9.6)	16 629 (23.4)
General	639 (5.1)	12 661 (32.0)		168 (1.5)	6 465 (18.3)		203 (2.1)	8 563 (24.0)		613 (6.5)	7 477 (19.8)
Rural residence	2768 (11.6)	56 257 (42.8)		786 (3.8)	28 192 (23.0)		1 101 (5.5)	38 685 (30.9)		2480 (12.2)	33 505 (26.7)
Having health insurance	94 (5.2)	20 674 (50.2)		27 (1.4)	12 284 (31.9)		34 (2.1)	15 578 (39.8)		98 (7.0)	12 578 (31.7)
Family size, no.	3364 (NA)	68 883 (NA)		967 (NA)	35 630 (NA)		1 293 (NA)	48 197 (NA)		3068 (NA)	41 929 (NA)
**Mother**											
Age, years	3064 (NA)	62 923 (NA)		881 (NA)	32 726 (NA)		1 190 (NA)	44 269 (NA)		2782 (NA)	38 334 (NA)
Education^e^											
No schooling	1380 (9.9)	18 141 (34.7)		318 (2.4)	8 236 (16.1)		542 (4.1)	11 933 (22.9)		1247 (9.6)	10 988 (21.1)
Primary school	611 (12.3)	10 627 (43.7)		184 (4.2)	5 380 (23.6)		214 (5.3)	7 380 (31.6)		564 (13.8)	6 438 (27.7)
Secondary school	1137 (9.9)	28 141 (43.8)		380 (4.2)	15 303 (25.8)		438 (5.3)	20 275 (33.9)		1035 (11.9)	17 126 (28.3)
High school or higher	236 (4.6)	11 974 (28.9)		85 (2.3)	6 711 (17.8)		99 (2.7)	8 609 (22.6)		222 (5.9)	7 377 (19.7)
**Child**											
Female sex	1583 (10.0)	31 896 (38.6)		443 (3.0)	16 565 (21.6)		591 (4.5)	22 345 (28.5)		1446 (10.6)	19 412 (24.7)
Age in months	3 348 (NA)	68 428 (NA)		961 (NA)	35 377 (NA)		1 286 (NA)	47 864 (NA)		3058 (NA)	41 611 (NA)
Birth order											
First	826 (8.7)	22 505 (38.0)		249 (3.0)	12 166 (22.0)		321 (4.3)	16 310 (28.9)		812 (10.1)	13 618 (23.7)
Second	1021 (10.3)	23 954 (40.2)		326 (3.8)	13 045 (23.5)		399 (4.9)	17 302 (30.6)		930 (11.2)	14 960 (26.5)
Third or more	1 517 (9.7)	22 424 (35.1)		392 (2.8)	10 419 (17.3)		573 (4.3)	14 585 (24.1)		1326 (10.0)	13 351 (22.3)

NA: not applicable.

^a^ Percentages were adjusted using sampling weights.

^b^ We analysed whether the service was received at all three phases in the care continuum: during pregnancy, during lactation and in early childhood.

^c^ Immunizations, pre-school education and growth monitoring.

^d^ Index of socioeconomic status was constructed by factor analysis using: household access to improved drinking water, improved latrine, clean cooking fuel, electricity, ownership of a house and land, housing materials for floor, roof and wall, and possession of 15 assets (including a mattress, pressure cooker, chair, bed, table, fan, television, sewing machine, phone, computer, refrigerator, watch, bicycle, motorbike and car) and livestock (cow, goat and chicken).

^e^ Education categories were grouped as follows for the total number of years of education attained by the individual: no schooling (0 years), primary school (1 to 5 years), secondary school (6 to 10 years) and high school or higher (≥ 11 years).

Notes: The total number of respondents were 35 570 and 185 101 in 2006 and 2016, respectively. For continuous variables the numbers represent the total number of individuals who received the service and had valid data for the respondent characteristics.

**Table 4:** Factors associated with use of supplementary food and nutrition counselling in the Integrated Child Development Services programme among pregnant and lactating women and their children in India, 2006 and 2016

Binary outcomes	OR (95% CI)				
	Supplementary food^a^			Counselling on nutrition^a^	
	2006 (*n* = 32 208)	2016 (*n* = 167 873)		2006 (*n* = 29 743)	2016 (*n* = 167 873)
**Household**					
Socioeconomic status index^b^					
Quintile 1 (poorest)	Ref.	Ref.		Ref.	Ref.
Quintile 2	1.02 (0.93 to 1.12)	1.04 (0.98 to 1.11)		0.98 (0.75 to 1.28)	1.13 (1.06 to 1.20)
Quintile 3	0.98 (0.86 to 1.12)	1.17 (1.05 to 1.29)		0.93 (0.70 to 1.24)	1.29 (1.16 to 1.44)
Quintile 4	0.76 (0.60 to 0.97)	1.06 (0.91 to 1.23)		0.82 (0.53 to 1.25)	1.27 (1.12 to 1.43)
Quintile 5 (richest)	0.34 (0.25 to 0.46)	0.89 (0.75 to 1.05)		0.43 (0.22 to 0.81)	1.10 (0.97 to 1.25)
Religion					
Hindu	Ref.	Ref.		Ref.	Ref.
Muslim	0.88 (0.65 to 1.20)	0.90 (0.82 to 0.98)		1.27 (0.91 to 1.77)	0.92 (0.83 to 1.02)
Christian	1.02 (0.63 to 1.66)	0.93 (0.83 to 1.00)		1.11 (0.66 to 1.88)	0.88 (0.73 to 1.06)
Other	1.54 (0.94 to 2.50)	0.92 (0.77 to 1.09)		1.37 (1.04 to 1.79)	1.00 (0.87 to 1.14)
Caste categories					
Scheduled castes	2.00 (1.52 to 2.63)	1.45 (1.30 to 1.62)		2.78 (2.04 to 3.80)	1.28 (1.15 to 1.42)
Scheduled tribe	2.02 (1.40 to 2.90)	1.37 (1.22 to 1.54)		3.45 (2.47 to 4.80)	1.21 (1.10 to 1.34)
Other backward classes	1.37 (0.95 to 1.97)	1.29 (1.18 to 1.41)		1.98 (1.16 to 3.39)	1.15 (1.04 to 1.28)
General	Ref.	Ref.		Ref.	Ref.
Rural residence	2.81 (1.97 to 4.01)	2.24 (1.86 to 2.69)		3.18 (1.83 to 5.50)	1.82 (1.52 to 2.16)
Having health insurance	0.81 (0.54 to 1.21)	1.24 (1.18 to 1.31)		0.56 (0.36 to 0.87)	1.18 (1.11 to 1.24)
Family size, no.	0.97 (0.94 to 1.00)	0.99 (0.98 to 1.00)		0.99 (0.95 to 1.03)	1.00 (0.99 to 1.01)
**Mother**					
Age, years	0.99 (0.99 to 1.00)	0.99 (0.98 to 0.99)		1.00 (0.98 to 1.02)	0.99 (0.98 to 1.00)
Education^c^					
No schooling	Ref.	Ref.		Ref.	Ref.
Primary school	1.19 (0.98 to 1.44)	1.13 (1.06 to 1.21)		1.49 (1.17 to 1.89)	1.12 (1.04 to 1.20)
Secondary school	1.21 (1.05 to 1.40)	1.16 (1.05 to 1.28)		2.05 (1.62 to 2.58)	1.16 (1.07 to 1.26)
High school or higher	0.86 (0.69 to 1.07)	0.84 (0.76 to 0.93)		1.81 (1.29 to 2.54)	0.94 (0.86 to 1.03)
**Child**					
Female sex	1.03 (0.90 to 1.17)	1.04 (1.00 to 1.07)		0.88 (0.80 to 0.98)	1.05 (1.01 to 1.08)
Age in months	1.00 (0.99 to 1.00)	1.00 (0.99 to 1.00)		1.00 (0.99 to 1.00)	1.00 (1.00 to 1.00)
Birth order					
First	Ref.	Ref.		Ref	Ref.
Second	1.22 (1.02 to 1.45)	1.17 (1.12 to 1.22)		1.35 (1.05 to 1.74)	1.14 (1.09 to 1.20)
Third or more	1.21 (1.04 to 1.41)	1.21 (1.13 to 1.28)		1.24 (0.91 to 1.69)	1.14 (1.08 to 1.21)

CI: confidence interval; OR: odds ratio; Ref: reference category.

^a^ We analysed whether the service was received at all three phases in the care continuum: during pregnancy, during lactation and in early childhood.

^b^ Index of socioeconomic status was constructed by factor analysis using: household access to improved drinking water, improved latrine, clean cooking fuel, electricity, ownership of a house and land, housing materials for floor, roof and wall, and possession of 15 assets (including a mattress, pressure cooker, chair, bed, table, fan, television, sewing machine, phone, computer, refrigerator, watch, bicycle, motorbike and car) and livestock (cow, goat and chicken).

^c^ Education categories were grouped as follows for total number of years of education attained by the individual: no schooling (0 years), primary school (1 to 5 years), secondary school (6 to 10 years) and high school or higher (≥ 11 years).

Notes: *n* is the total number of respondents. As some data were missing for the mother’s and the child’s age we used data only for the set with all complete variables. The following 12 outcomes of integrated child development services were analysed over three phases in the care continuum. During pregnancy: (i) supplementary food; (ii) health and nutrition education; (iii) health check-ups. During lactation: (iv) supplementary food; (v) health and nutrition education; (vi) health check-ups. During early childhood: (vii) supplementary food as take-home rations or hot cooked meal; (viii) health check-ups; (ix) nutrition counselling for the mother after the child was weighed; (x) childhood immunizations; (xi) early childhood care and preschool education; and (xii) growth monitoring. We adjusted estimates are for sampling weights and standard errors clustered at the state level. All specifications include state-fixed effects (*N–*1 dummy variables for 35 Indian states).

**Table 5:** Factors associated with use of health check-ups and child-specific services in the Integrated Child Development Services programme among pregnant and lactating women and their children in India, 2006 and 2016

Binary outcomes	OR (95% CI)				
	Health check-ups^a^			Child-specific services^b^	
	2006 (*n* = 29 795)	2016 (*n* = 167 873)		2006 (*n* = 32 208)	2016 (*n* = 167 873)
**Household**					
Socioeconomic status index^c^					
Quintile 1 (poorest)	Ref.	Ref.		Ref.	Ref.
Quintile 2	1.04 (0.86 to 1.26)	1.11 (1.04 to 1.18)		1.11 (0.98 to 1.25)	1.03 (0.93 to 1.14)
Quintile 3	0.99 (0.85 to 1.15)	1.28 (1.15 to 1.43)		1.12 (1.01 to 1.25)	1.17 (1.01 to 1.36)
Quintile 4	0.73 (0.53 to 1.02)	1.23 (1.06 to 1.42)		0.80 (0.56 to 1.14)	1.16 (1.01 to 1.33)
Quintile 5 (richest)	0.40 (0.24 to 0.67)	1.07 (0.90 to 1.26)		0.52 (0.33 to 0.81)	0.97 (0.81 to 1.15)
Religion					
Hindu	Ref.	Ref.		Ref.	Ref.
Muslim	0.90 (0.67 to 1.21)	0.95 (0.85 to 1.05)		0.98 (0.70 to 1.37)	0.93 (0.84 to 1.04)
Christian	1.12 (0.63 to 2.01)	0.93 (0.83 to 1.05)		0.92 (0.76 to 1.12)	0.87 (0.76 to 1.00)
Others	1.78 (1.34 to 2.38)	0.83 (0.66 to 1.04)		1.39 (0.76 to 2.56)	1.10 (0.96 to 1.27)
Caste categories					
Scheduled castes	2.04 (1.51 to 2.76)	1.35 (1.21 to 1.52)		1.73 (1.30 to 2.31)	1.42 (1.32 to 1.53)
Scheduled tribe	2.46 (1.77 to 3.43)	1.37 (1.21 to 1.56)		1.80 (1.40 to 2.30)	1.35 (1.21v1.51)
Other backward classes	1.62 (1.11 to 2.38)	1.22 (1.10 to 1.36)		1.35 (0.97 to 1.89)	1.23 (1.16 to 1.31)
General	Ref.	Ref.		Ref.	Ref.
Rural residence	3.91 (2.35 to 6.49)	2.01 (1.67 to 2.42)		2.92 (1.82 to 4.70)	1.88 (1.58 to 2.22)
Having health insurance	0.78 (0.47 to 1.29)	1.20 (1.13 to 1.28)		0.87 (0.59 to 1.27)	1.10 (1.02 to 1.19)
Family size	1.00 (0.97 to 1.03)	1.00 (0.98 to 1.01)		0.99 (0.96 to 1.01)	1.00 (0.99 to 1.02)
**Mother**					
Age in years	1.00 (0.99 to 1.02)	0.99 (0.98 to 1.00)		1.00 (0.98 to 1.01)	0.99 (0.99 to 1.00)
Education^d^					
No schooling	Ref.	Ref.		Ref.	Ref.
Primary school	1.15 (0.92 to 1.45)	1.12 (1.05 to 1.19)		1.22 (1.05 to 1.42)	1.16 (1.10 to 1.23)
Secondary school	1.52 (1.25 to 1.84)	1.17 (1.05 to 1.30)		1.27 (1.04 to 1.56)	1.11 (1.03 to 1.21)
High school or higher	1.25 (1.01 to 1.57)	0.88 (0.78 to 1.00)		0.91 (0.69 to 1.22)	0.89 (0.81 to 0.99)
**Child**					
Female sex	1.00 (0.89 to 1.12)	1.03 (0.99 to 1.07)		1.04 (0.94 to 1.16)	1.05 (1.00 to 1.10)
Age in months	1.00 (0.99 to 1.00)	1.00 (0.99 to 1.00)		1.00 (0.99 to 1.01)	1.01 (1.00 to 1.01)
Birth order					
First	Ref.	Ref.		Ref.	Ref.
Second	1.14 (0.91 to 1.44)	1.14 (1.08 to 1.20)		1.11 (0.96 to 1.28)	1.15 (1.10 to 1.20)
Third or more	1.12 (0.88 to 1.41)	1.16 (1.08 to 1.25)		1.12 (0.98 to 1.28)	1.15 (1.08 to 1.23)

CI: confidence interval; OR: odds ratio; Ref: reference category.

^a^ We analysed whether the service was received at all three phases in the care continuum: during pregnancy, during lactation and in early childhood.

^b^ Immunizations, pre-school education and growth monitoring.

^c^ Index of socioeconomic status was constructed by factor analysis using: household access to improved drinking water, improved latrine, clean cooking fuel, electricity, ownership of a house and land, housing materials for floor, roof and wall, and possession of 15 assets (including a mattress, pressure cooker, chair, bed, table, fan, television, sewing machine, phone, computer, refrigerator, watch, bicycle, motorbike and car) and livestock (cow, goat and chicken).

^d^ Education categories were grouped as follows for total number of years of education attainted by the individual: no schooling (0 years), primary school (1 to 5 years), secondary school (6 to 10 years) and high school or higher (≥ 11 years).

Notes: *n* is the total number of respondents. As some data were missing for the mother’s and the child’s age we used data only for the set with all complete variables. The following 12 outcomes of Integrated Child Development Services were analysed over three phases in the care continuum. During pregnancy: (i) supplementary food, (ii) health and nutrition education, (iii) health check-ups. During lactation: (iv) supplementary food, (v) health and nutrition education, (vi) health check-ups. During early childhood: (vii) supplementary food as take-home rations or hot cooked meal, (viii) health check-ups, (ix) nutrition counselling for the mother after the child was weighed, (x) childhood immunizations, (xi) early childhood care and preschool education and (xii) growth monitoring. We adjusted estimates for sampling weights and standard errors clustered at the state level. All specifications include state-fixed effects (*N*–1 dummy variables for 35 Indian states).

Caste differences in use of services appeared to change over time, holding wealth constant. In 2006, the odds of receiving supplementary foods was twice as high among scheduled castes (OR: 2.00, 95% CI: 1.52 to 2.63) and scheduled tribes groups (OR: 2.02, 95% CI: 1.40 to 2.90) compared with general castes. In 2016, the differences were smaller (OR: 1.45, 95% CI: 1.30 to 1.62 and OR: 1.37, 95% CI: 1.22 to 1.54, respectively). Similar findings were seen for the other outcome measures.

Similar to caste, differences in service use across education levels also changed over time. For example, women with primary or secondary schooling in 2006 had higher odds of having counselling during pregnancy (OR: 1.49, 95% CI: 1.17 to 1.89 and OR: 2.05 95% CI: 1.62 to 2.58, respectively) than women with no education. By 2016, the odds had reduced (OR: 1.12, 95% CI: 1.04 to 1.20 and OR: 1.16, 95% CI: 1.07 to 1.26, respectively). Again, while statistical significance varied, the trend of a reduction in the odds ratios across education levels was similar for all outcomes.

Having health insurance, which few households had access to before 2008, was negatively associated with the use of nutrition counselling in 2006 (OR: 0.56, 95% CI: 0.36 to 0.87). However, in 2016 those with health insurance had higher odds of receiving all services (OR: 1.24, 95% CI: 1.18 to 1.31 for supplementary food; OR: 1.18, 95% CI: 1.11 to 1.24 for nutrition counselling; OR: 1.20, 95% CI: 1.13 to 1.28 for health check-ups and OR: 1.10, 95% CI: 1.02 to 1.19 for child-specific services). Finally, female children (OR: 1.05, 95% CI: 1.00 to 1.10) and second- and third-born children (OR: 1.15, 95% CI: 1.10 to 1.20 and OR: 1.15, 95% CI: 1.08 to 1.23, respectively) had slightly higher odds of using early childhood services.

In our robustness checks, we found similar findings when using the 12 individual services as outcomes (data repository),^17^ and when using the composite coverage indicators by child’s age group. 

Finally, we saw that trends and determinants of service use in Uttar Pradesh and Bihar did not differ substantially from those for overall national data (data repository).^17^ We found higher odds ratios for use of all services among scheduled caste groups compared with other groups in both 2006 and 2016.

## Discussion

Using two nationally representative data sets we provide evidence on how the use of India’s Integrated Child Development Services programme has changed in the decade after reform of the programme. India appears to be well on its way to scaling-up of nutrition-specific interventions using the integrated services. This large expansion in services is laudable given challenges such as decentralization of implementation to the state level, high numbers and diversity of the population, constraints on funding, and lack of community awareness, among others.^6^ Indeed, these challenges are reflected in our findings that expansion in use of services varied considerably at the state level and by sociodemographic characteristics. Even though households in the poorest quintile were better reached by the services in 2015–2016, the wealth inequality in use widened over the decade studied.

The exclusion of the poorest people from services is concerning. Most of the poor who were left behind were from states known to be weak performers on the programme, such as Uttar Pradesh^22^ and Bihar,^23^ suggesting that overall poor performance in high-poverty states could lead to major exclusions. The exclusion of the poorest quintile could also be due to the challenges of reaching remote or difficult geographical areas, even in better-performing states, despite attempts to close equity gaps district-by-district.^24^ There can also be local challenges of exclusion within villages due to caste or location.^25^ Further investigations around the potential reasons for exclusions could help in addressing inequity gaps.

The risk of exclusion of the poorest households has also been documented in the coverage of India’s safe motherhood cash-transfer programme, the *Janani Suraksha Yojana*.^26^^,^^27^ Even in a state such as Odisha, with a well-performing health system, similar inequitable patterns of use were seen for a conditional cash-transfer programme for nutrition and health.^28^ Some authors have attributed the exclusion of the poorest households in certain programmes to difficulties in complying with the programme conditions.^29^ It is also unlikely that the use of services has many barriers that prevent poorer people from using services that are locally available. Except for entry-level barriers, such as pregnancy registration, the unconditional nature and universal scope of the services should make it accessible to all. As noted above, a more plausible explanation is weak performance of service implementation in states or districts with the highest proportions of poorer people. Indeed, in the two states of Uttar Pradesh and Bihar, we found greater exclusion by caste as well, suggesting that exclusions in these states is due to overall poor performance, leading to low coverage for all, rather than targeted exclusions for some. In separate policy-focused descriptive analyses of coverage,^30^ we provide district-specific coverage estimates for all programme services that can inform policy-makers in India and elsewhere. 

Despite lingering caste discrimination in India,^31^ it is reassuring that caste and tribe-based exclusion from the programme services has declined. The caste differences appear to favour the traditionally marginalized scheduled castes and scheduled tribes groups compared with the general castes, after controlling for wealth. In Odisha and in Chhattisgarh, where there are large pockets of tribal populations, efforts to strengthen overall programme services with a view to improving equity of access^10^ has likely helped close gaps for tribal communities. In Maharashtra, a targeted focus on tribal areas as part of the state nutrition mission^32^ has also likely helped to close some gaps.

Despite the hypothesis that girls are discriminated against in health service use in India,^33^ we did not find evidence of son preference in families’ use of programme services. Instead, we found that being a female child significantly improved the likelihood of receiving the range of services, although the difference was slight. Similarly, there was no indication that children who were higher in birth order had lower use of services.

Health insurance coverage improved 10-fold between 2006 and 2016, likely due to the introduction of a national health insurance programme, the *Rashtriya Swasthya Bima Yojana*, for households below the poverty line in 2008. This programme is currently operational in 25 states. In our study, those with health insurance were about twice as likely to use the services in 2016. However, since these services are free of charge there is no direct role for insurance in gaining access. This finding could reflect self-selection; households that take up government health and nutrition services are also more likely to be enrolled in the national health insurance. Also, the same states could perform better both on the expansion of the national health insurance programme and on child development services.

Our study had several strengths. It offers an in-depth analysis of individual and household access to a large, universally-offered nutrition programme that targets the first 1000 days of life. The study examines the inequity in the expansion in use of the programme and investigates the complex factors associated with use. By comparing the coefficients of these covariates over time, we have documented how the child development services in India have become more inclusive between 2006 and 2016 and yet, how some groups, such as the poorest wealth quintiles, are still not being adequately served. Finally, our results are robust to several alternative regression specifications, including adjustments for state performance.

Although the survey questions remained the same over time (thus enabling comparisons), they are limited to participation in the last 12 months for children or during pregnancy and lactation. Finer-grained questions could enquire about the regularity and intensity of participation, the actual consumption of foods provided through the programme and the regularity of the use of specific services, such as growth monitoring. Such questions could help planners and policy-makers make more informed assessments about strengthening programme services.

Instead the study focused on changes over time, it is not longitudinal at the individual level. Our ability to analyse the intensity of participation in the programme is limited by the availability of the survey questions included in India’s national family health survey questionnaires. Finally, an impact assessment of the programme on maternal and child health is beyond the scope of this paper and is an important area for future research. The post-reform period of the programme coincided with a period that also saw a 10-percentage point reduction in stunting among children aged 0 to 5 years.^34^

India’s policy reforms have increased coverage of the Integrated Child Development Services programme at a national level and reached marginalized groups. With further scaling-up, the programme needs to focus on effective subnational implementation to reach households from the lowest socioeconomic strata and women with low schooling levels, as these high-need groups are currently more excluded in India.
